# Understanding an aorto-atrial fistula in a patient with heart failure

**DOI:** 10.1007/s12471-026-02023-z

**Published:** 2026-02-02

**Authors:** Rita Almeida Carvalho, Débora Sá, Pedro Magro, Marisa Trabulo, Miguel Mendes, Regina Ribeiras

**Affiliations:** 1 Cardiology Department, Hospital de Santa Cruz, Unidade Local de Saúde Lisboa Ocidental, Lisbon, Portugal; 2 Cardiology Department, Hospital Dr. Nélio Mendonça, Funchal, Portugal; 3 Cardiac Surgery Department, Hospital de Santa Cruz, Unidade Local de Saúde Lisboa Ocidental, Lisbon, Portugal

A 37-year-old previously healthy man presented with progressive dyspnea, peripheral edema, and weight gain over four months. Examination revealed a continuous heart murmur with pulmonary and peripheral congestion. NT-proBNP was markedly elevated despite high-dose furosemide therapy.

Transthoracic and three-dimensional transesophageal echocardiography (3D TOE) identified an aorto-atrial fistula from the non-coronary sinus of Valsalva to the right atrium (Fig. 1a, yellow arrows; Supplementary Videos 1–4). The fistulous jet coursed parallel to the tricuspid annulus, impinging eccentrically on the right atrial wall. A concomitant 16-mm ostium secundum atrial septal defect produced a significant left-to-right shunt (Fig. 1a, green arrow; Supplementary Videos 2–3). No evidence of endocarditis was found, and the fistula was considered congenital.

**Fig. 1:**
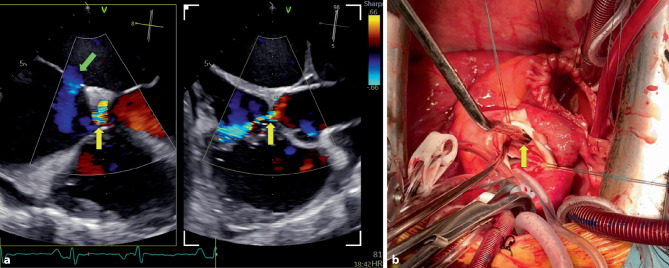
**a** Transesophageal echocardiography with color Doppler demonstrating an aorto-atrial fistula from the non-coronary sinus of Valsalva to the right atrium (*yellow arrows*) and a concomitant ostium secundum atrial septal defect (*green arrow*). **b** Intraoperative view showing the fistulous pathway (*yellow arrow*) during surgical repair

The patient underwent successful surgical repair of both defects (Fig. 1b, yellow arrow showing the fistulous pathway) and was discharged uneventfully. This rare case highlights the crucial role of multimodality imaging in guiding tailored surgical planning.

## Supplementary Information

ESM1: Supplementary material 1

ESM2: Supplementary material 2

ESM3: Supplementary material 3

ESM4: Supplementary material 4

